# Harmful effects of shisha: literature review

**DOI:** 10.1186/1755-7682-7-16

**Published:** 2014-04-04

**Authors:** Hafiz Muhammad Aslam, Shafaq Saleem, Sidra German, Wardah Asif Qureshi

**Affiliations:** 1 Dow Medical College, Dow University of Health Sciences, Karachi, Pakistan

## Abstract

Tobacco is a preventable cause of morbidity and mortality across the world. A recently infamous way of smoking tobacco is shisha. Shisha smoking is also known as water pipe, hookah and Narghile smoking. The percentage of shisha smokers is on the rise rapidly spanning the globe. A literature review was conducted to identify all evidence on the epidemiological variations and health effects of shisha smoking. “Pub med” is used as a searching tool to identify all relevant empirical studies conducted worldwide. A qualitative overview of evidence is presented.

Exposure to Shisha smoking is significantly associated with low infant weight, heart rate variations, hyperglycemia and hypertriglyceridemia. Increased risk of carcinoma is also leagued with it including carcinomas of the pancreas and lung being at the forefront. In conclusion, this review identifies grounds of several adverse conditions being associated with the habit of shisha smoking. It also evaluates the relevant epidemiological variations around the globe. The review culminates in the importance of enlightening shisha smokers regarding its deleterious effects.

## Findings

### Introduction

Tobacco is a preventable cause of morbidity and mortality across the globe. Low and middle income countries are the most severely affected. Tobacco-attributable deaths are projected to decline by 9% between 2002 and 2030 in high-income countries, but to double from 3.4 million to 6.8 million in low- and middle-income countries [1]. Tobacco use is responsible for about 5 million deaths per year world wide Furthermore, it is a fact that half of the people who smoke today will die prematurely [2]. Tobacco use is the 2nd major leading cause of death and is currently responsible for the deaths of one in ten adults’ across the world [3]. At present, the use of Shisha to smoke tobacco is contributing at large scale in its increased use, a practice dating back at least 400 years [2]. These projections also bring to light the need to study the trends and pattern of tobacco usage in different forms [4].

Shisha is also known as Narghile, hookah, Hubble bubble and water pipe in different cultures and countries. It is a way of smoking tobacco in which the vapor passes through water before inhalation [4]. At present, Shisha is becoming an increasingly popular way of tobacco use world wide. It takes its roots from Eastern Mediterranean region, and is now gaining popularity in western countries including Australia, UK, Canada and USA [5]. Present day shisha smoking masses include the youth mainly university and college students and also high school children [2].

Recently shisha has been considered as a global threat and given the status of an epidemic by public health officials. Tobacco smoke contains over 4800 different chemicals out of which 69 are carcinogens and several others are tumor promoters [6,7]. It is popular due to the common misconception that the nicotine content in shisha is lower than that of cigarettes and that water used in this form of tobacco intake works as a filter, removing all the hazardous chemicals such as CO, nicotine and tar. These common misapprehensions lead public to believe that shisha smoking is not a hazard their health and others’ [4].

The purpose of this review remains to describe that tobacco smoking using Shisha is emerging as a “virulent strain” in the tobacco “epidemic”. The review thus focuses on research related to water pipe epidemiology, health effects and public policy.

### Methodology

#### Terminology and description

The different terms used to describe Shisha vary depending upon different regions and include ‘water pipe’ ‘bory’ or ‘gaza’ in Egypt, Saudi Arab and Pakistan. In Jordan, Lebanon and Syria its known as ‘Narghile’ ‘Nargile’ or ‘arghile’ , while Hookah in the Indian Subcontinent. There is regional variation in shape, size, appearance and the kind of tobacco smoked. In this study Shisha or Hookah refers to the kind of tobacco smoking in which the smoke or the vapor passes through water.

#### Search strategy and selection criteria

The objective of our review is to focus upon epidemiological variation and the harmful effects of shisha. The general protocol of locating relevant studies spanned multiple sources which include the search engines of “Pub med” and “Google scholar” by employing the terms “shisha”, ‘water pipe’ , “narghile’ and “hookah”. Using the word “Shisha” in Pub med, 91 articles were found from 1994–2012, term “Narghile” turned up 94 articles from 2013 to 1981, the word “hookah” unearthed 146 articles from 2013–1982 and the term “water pipe” brought to front 186 articles from 1986–2013. All search results including original articles, case reports or reviews in English language were included. Out of them the ones regarding epidemiology and harmful effects of shisha were sieved. Exclusion criteria are to bar those articles which are not apt to the purpose and objective of article.

### Discussion and result

#### Epidemiology around the globe

##### In United States of America

The prevalence of Shisha smoking is peaking in United States as it reaches up to 40% from 2005 – 2008 [8]. A Study conducted in two universities of United States reveals the prevalence of life time hookah users as 27.8% [5]. Another survey conducted at the University of San Diego unveiled that the prevalence of shisha smoking to be 24.5% [8]. In yet another study it is reported that out of the whole sample there are more than 1/3rd (40.3%) of people who have ever smoked tobacco from a shisha [9]. Maziak W reports that shisha smoking is continuing to spread among populations worldwide and perhaps represents the second global epidemic since that of cigarette; the prevalence being 6-39% [10]. Braun RE states in his study that 15.4% students had previously smoked shisha and 6% did so within past 30 days [11]. In another analysis conducted at Wayne University, prevalence of shisha smoking was 10% in students and while another study evaluated it to be 26% [12,13]. Jordan HM reports prevalence of 9.7% among the three thousand and ten New Jersey high school students while Grekin ER unearths that 15% of students had used Shisha once in their life time [14,15] (Table 1).

**Table 1:** Represents studies regarding prevalence, gender distribution, reason for smoking and misconception

**Study**	**Country**	**Study population**	**Type of study**	**Sample size**	**Prevalence**	**Mean age**	**Gender distribution**	**Reason for smoking**	**Misconception of shisha smoking is better than cigarette smoking**
Brockman NL et al. [5]	United States	**Students of two large universities**	**Cross-sectional**	**307 facebook profile owner**	60(27.8%)	18.8 ± 0.6	Male = 31(51.7%)	-	-
							Female = 29(48.3%)		
Sutfin EL et al. [9]	United States	**Students of eight universities**	**Cross sectional**	**3770**	40.3%	17.9 ± 1.6	Male = 328(50%)	-	32%
							Female = 325(50%)		
Braun RE [11]	United States	**University studens**	**Cross sectional**	**2000**	15.4%	23.1 ± 12.32	Male = 40%	Socializing/partying = (29%), peer influence = (27%), relaxation (25%)	
							Female = 60%		
Taha AZ et al. [16]	Saudi Arabia	**Students of three colleges.**	**Cross sectional**	**500 male students**	12.6%	6 to 18 years	Study only on male students	-	-
Jawaid A et al. [17]	Pakistan	**Students of Four univerisities**	**Cross sectional**	**450 participants**	241(53.6%)		Male = 268(59.6%)	Curiosity = 61.4%	269(60%)
							Female = 182(40.4%)	Pleasure seeking = 46.9%	
								Peer pressure = 22.8%	
								Boredom = 17.8%	
								Stress = 10.8%	
Jaffri SB [18]	Pakistan	**Students of college and universities**	**Cross sectional**	**422 students**	90(49%)	21.6	Male = 30%	Curiosity = 42.9%	29(7.1)
							Female = 15.2%	Pleasure seeking = 33.1%	
								Boredom = 18.4%	
								Previous smokers = 14.6%	
								Peer pressure = 14.6%	
								Stress = 10.3%	
Rehman S [4]	Pakistan	**Four cities**	**Cross sectional**	**406 participants**	246(61%)	22.27 ± 4	Male = 175(71%)	Curiosity = 61%	282(69%
							Female = 71(29%)	Social trends = 55%	
								Peer pressure = 57%	
Gadalla S [19]	Qualyobia governorate	**Two rural secondry schools**	**Cross sectional**	**635 students**	19%	12	Male = 26%	Smokers have more friends = 34% of males, 26% of the females.	-
							Female = 5%		
								Smoking makes boys look handsome = 8% of males, 3% of females	

### Non –US prevalance

#### In Pakistan

Very few studies have been conducted in order to assess the prevalence of Shisha in Pakistan. A study encompassing medical and dental students of Karachi reveals 22.7% students are shisha smokers [20]. In another study carried out at Aga Khan University, Shisha prevalence is found out to be 53.6% [17]. The surveys conducted at the Institute of Business and Administration, the Preston University and Karachi University reveal that the prevalence of current smokers there is 49% (3.3% smoke daily, 7.1% 1–2 times/week, 38.6% occasionally on gathering) [18]. A cross sectional study executed in four large cities of Pakistan discloses the prevalence of 61% shisha smokers who predominantly smoked shisha occasionally [4]. In a study conducted at small semi urban community of Karachi, it was evaluated that 19% of tobacco smokers were shisha users [21] (Tables 1 and 2).

**Table 2:** Represents studies regarding prevalence, gender distribution, reason for smoking and misconception

**Serial no**	**Study**	**Country**	**Prevalence**	**Study population**	**Type of study**	**Sample size**	**Mean age**	**Gender distribution**	**Reason for smoking**	**Misconception of shisha smoking is better than cigarette smoking**
1	Amin TT et al. [22]	Egypt	53.9%	**Secondry school students**	**Cross sectional**	**1,652**		Male = 30.3%	1) more socially acceptable than cigarettes (52.1%)	49.7%
								Female = 8.5%		
									2) relieve stress and tensions (37.8%)	
2	Israel E et al. [23]	Egypt		**Cafe workers**	**Cross sectional**	**-**	20	-	-	-
3	Al-Naggar RA [24]	Malaysia	20%	**Medical students**	**Cross sectional**	**300**	22.5 ± 2.5	-	-	-
5	Maziak W et al. [25]	Syria	-	**University students**	**Cross sectional**	**587**	Male = 19.2 ± 2.2	Male = 62.6%	-	-
							Female = 21.7 ± 3.2	Female = 29.8%		
6	Braun RE [11]	USA	15.4%	**University students**	**Cross sectional**	**2,000**	23.1 ± 12.32	Male = 40%	Socializing/partying = (29%), peer influence = (27%), relaxation (25%)	-
								Female = 60%		
7	Aljarrah k et al. [26]	Jordan	24.4%	**Hookah users of cafe of san diego down town**	**Cross sectional**	**235**	17-35 years	Male = 32(24.2%)		58.3%
								Female = 24(24.5%)		
8	Dar-Odeh NS [27]	Jordan	Male = 21(106), Female = 53 (63%)	**Three jordanian universities**	**Cross sectional**	**1454**		Male = 21(106), Female = 53(63%)		89.06

### Worldwide prevalance

Prevalence of Shisha smoking is on the rise Worldwide. In Lebanon, the global youth tobacco survey conducted in 2005 included 13–15 years old, it concluded that 59.8% of them had smoked shisha at least once in the past month as opposed to only 10% of them being cigarette smokers [28].

Studies spanning three different colleges of Saudi Arabia indicate the prevalence of 12.6% shisha users [16]. Study conducted in two rural schools of Qualobia and Management and Science University in Malaysia unveiled the prevalence of Shisha smokers being 19% and 20% [19,24]. In a study at Beirut, which was conducted on adolescents of age 13–20 years, it was evaluated that 60% of respondents had smoked shisha at least once in their life time, while a study in Syria including University students, it was evaluated that 62.6% male and 29.8% female were regular smokers of Shisha [25,29] (Tables 1 and 2).

### Gender and age distribution

A Study held in two large universities of the United States shows that shisha smokers are mostly males with ages between 15–25 years [5]. In Saudi Arabia the scenario is similar as 63.8% students there start smoking shisha at ages of 16–18 years the male gender being dominant [16]. In Syria mostly the age of commencement of this habit is found to be 19.2 years for males and 21.7 years for females, on an average [25]. Conditions in Pakistan were found not to be vary much. Jawaid A et al. reports males as dominant users of Shisha (53.6%) [17]. Jaffary SB et al. also reports male predominance in shisha smokers having a mean age of 21 years [18], same male dominance and age group were reported by Rehman S [4]. Surveys conducted in all Middle Eastern countries also reveal that males predominate the Shisha-users quo. Similar findings were also indicated in a study done in Egypt and Qualabia [19,22,25,30]. However in Jordan the trend was found to be totally opposite as more females are found smoking shisha as compared to males [27].

### Reason for shisha smooking

The 15 Reasons of shisha smoking

1. Global Tourism and Migration Flows (back from Egypt, Tunisia, etc. with a hookah in the suitcase; hookah lounges in the West)

2. A New Hassle-Free Lighting System (new easy to light charcoal)

3. Relative Acceptance by Non-Smokers (notable smoke irritants filtered out)

4. Unexepected Backlash Effect of Anti-Tobacco Campaigns (viewed as safer than cigarette smoking)

5. Filtration of Some Noxious Substances (some carcinogens, among others, may be filtered out)

6. A “Light” Dependence (seen as easy to quit)

7. The Influence of Television (case of the Arab World) (Egyptian movies have featured hookah smokers for decades)

8. The Rise of Individualism in Modern Societies (socialising needs and the search for new forms of sociability)

9. Conviviality (“social” smoking, sharing the hose (ludens), talking, long time passing)

10. 10, A Powerful Symbolism (dream, art, “mysticism”, “peace pipe”)

11. A Transverse Social, Sexual, Religious and Inter-Generational Practice (social and cultural melting pot)

12. Flavours (“tobamel” (muassel), a flavoured tobacco (or no-tobacco)-honey/molasses based mixture)

13. The Cultural Status of Honey (Koran, The Bees)

14. A Highly Sensory Experience (Five senses permanently stimulated)

15. “Rebellion” Values [31]

### Attitudes and beliefs regarding shisha use

Attitudes and belief about tobacco use are the singular main factors affecting people’s behavior towards shisha use. Till now very few studies have investigated attitudes and beliefs towards shisha use. Markets in even the developed countries promote the fallacy shisha is less hazardous than smoking and it is the main driving factor behind its current popularity. Regrettably very few studies have addressed this issue. Out of the few relevant studies conducted one states that, 30% of university students believe in the fallacy about shisha being less deleterious than cigarette [32]. In a study in Egypt 21% out of 206 male shisha smokers reported that they preferred Shisha for the similar belief. A study in Pakistan evaluated that 60% of population considers cigarettes to be more deleterious [4]. Same concept was also found in a study in Egypt, Malaysia and Jordan [23,24,33].

Research however has proven otherwise, suggesting three additional risks to health of water pipe smoking over cigarette smoking. The first being this as shisha is smoked over coal this adds to the already many harmful toxins to the smoke possesses. Secondly, a shisha smoker inhales up to 200 times more smoke in a single session as compared to cigarette smokers. Thirdly it is linked to high rates of second hand smoking due to its high social acceptance. (Tables 1 and 2)

The famous misperception that the shisha is filtered due to the water in it seems to be one main belief justifying it being less injurious, however it is well known that making air bubbles pass through water doesn’t change their content and since the volatile carcinogen of tobacco smoke and other particles will stay within the air bubbles during their passage through water, it does not render shisha any less harmful, at least, than cigarette [26].

### Comparison of shisha smoke with cigrette smoke

With a smoking regimen consisting of 171 puffs each of 0.53 l volume and 2.6 s duration with a 17 s inter-puff interval, the following results were obtained for a single smoking session of 10 g of mo'assel tobacco paste with 1.5 quick-lighting charcoal disks applied to the narghile head Shisha.

The smoke contained 2.94 mg Nicotine, 802 mg tar, 145 mg CO and relative to a smoke of single cigarette, greater quantities of chrysene, phenanthrene and flouranthrene [34]. It is also a fact that number of puffs and their volume from using shisha are about 10 times higher than cigarette, and higher concentration of metals while burning temperature for shisha is about 900°C as compared to 450°C for cigarette [26].

Peak concentration of nicotine in cigarette and shisha are same but the relatively long duration of the shisha use result in considerably greater effective nicotine exposure. Using a single-compartment pharmacokinetics model with linear clearance kinetics, and a nicotine clearance constant of 0.0333 min–1 obtained by fitting an exponential decay curve to the average nicotine concentrations at 5, 15, 30, and 45 minutes post–cigarette initiation (R2 = 0.98), the nicotine AUC observed in this study was 243 ng/ml-min for the cigarette and 418 ng/ml-min for shisha. Relative to a cigarette, shisha smokers were exposed to 1.7 times the nicotine dose when they were smoking tobacco through shisha [35].

### Cardiovascular effects

The injurious effects of Shisha smoking also encase the cardiovascular system and after 45 minutes of shisha use, heart rates are found to be significantly increased [10]. Many studies report that a mean increase in systolic and diastolic blood pressure and heart rate of shisha smokers is observed after shisha smoking [3,35-37]. Unlike cigarette smoking, little is known about health effects of shisha use. One acute effect is dysfunction in autonomic regulation of the cardiac cycles, as reduction in heart rate variability. Reduced heart rate variability is associated with inhalation exposure induced oxidative stress and increase in heart rate and blood pressure [3,35-37]. Numair KA reported that serum concentration of HDL, Apo A in shisha smokers were significantly lower than non smokers. However LDL-cholesterol, Apo B and triglycerides were significantly higher in smokers [3].

### Antioxidant and vitamin C

In a study conducted in Saudi Arabia, antioxidant capacity was evaluated by collection of two overnight blood samples; results indicated that total antioxidant capacity and vitamin C levels were lower in smokers than in non smokers [3].

### Effect on platelet function

Wolfram RM reported that single shisha smoking session increases oxidation injury (8-epi-PGF2 alpha p = 0.003), MDA(p = 0.001) and 11 DH = TXB2 (p = 0.0003) significantly. It was also reported that daily smoking induces persistent long lasting oxidation injury [38].

### Effect on pregnancy outcome

Female smoking prevalence differs greatly from country to country. It depends on educational and cultural atmosphere. It has been evaluated that smoking one or more shisha per day during pregnancy is associated with at least 100gm reduction of weight. Also that risk of delivering low birth babies almost triples among those who take up the practice of shisha smoking shisha in 1st trimester [39]. Tamim H reports that mothers who smoke shisha had low birth weight infant compared with non smoking mother (OR = 2.4, 95%CI, 1.2-5) [40]. In addition to these other problems are also associated with shisha smoking such as lower APGAR score and increased pulmonary problems at birth [39].

### Plasma nicotine concentration and effect on respiratory rate

Eissenberg T reports that for a shisha smoker, mean pre-smoking plasma nicotine concentration was 2.0 ± 0.2 ng/ml that increased to 6.1 ± 1.1 ng/ml at 5 minutes, 6.4 ± 0.8 ng/ml at 15 minutes,7.9 ± 1.0 ng/ml at 30 minutes, and 8.5 ± 1.0 ng/ml at 45 minutes [t(30) > 3.6; Ps < 0.001]. The higher nicotine associated with waterpipe tobacco smoking relative to cigarette smoking was significant at 45 minutes [t(30) = 4.3; P < 0.001] [27,35]. Mean CO Hb is increased by 4 times for starting 5 min of shisha smoking than by entire cigarette [9]. Respiratory rate also increases upto 2+/−2 breathes/min. [36]. A single session of shisha smoking produces four to five times higher CO than that produced from smoking a cigarette. When breath of heavy tobacco smoker was measured, CO level of 30-40 ppm were found. These levels indicate that approximately 5-7% of blood was not functioning properly. Breath of Shisha smoker measure 40–70 ppm of CO resulting in 8-12% of blood being effected [41]. One serve of hookah smoking caused elevation of mean CO value by almost eight folds higher than that of cigarette smoking [42,43].

### Serum nitric oxide concentration

Ghasemi A reports in his case control studies that in shisha smokers serum NO is much higher than in non-smokers [34.3 micromole/L (95%CI 27.8-42.3) vs 22.5 micromole/L (95%CI 18.4-27.6) [44].

### Effect on larynx

Case control study carried out in Beirut reports that incidence of benign lesion of the vocal cords and vocal folds in the shisha smoking group is 21.5% with edema being the most common presenting 16% of the time, followed by cyst presenting at 4.8% [45].

### Metabolic syndrome

Shisha smokers are found to be significantly more likely to have hypertryglycedemia (OR 1.63, 95%CI, 1.25-2.10), Hyperglycemia (OR 1.82, 95%CI, 1.37-2.41), Hypertension (OR 1.95, 95%CI, 1.51-2.51) and abdominal obesity (OR 1.93, 95%CI, 1.52-2.45) [46].

### Oral effects

Shisha smoking has many negative effects on mouth including staining of teeth, dental restorations and reduced ability of smell and taste. Shisha is one of the notable risk factor for periodontal bone loss, dry sockets and oral squamous cell carcinoma [27,47].

### Esophageal carcinoma

Although cigarette smoking is an established risk factor for esophageal carcinoma, there is little information regarding its association with Shisha. Dar NA reports that there is significant association between esophageal carcinoma and Shisha Smoking (OR = 1.85, 95%CI, 1.41-2.44) [48]. Its association has also been reported in a studies conducted at China, India and Iran [41,49,50].

### Pancreatic cancer

Cancer of the pancreas is a rapidly fatal disease and it significantly associated with tobacco smoking [51]. The disease is caused by damage (mutations) to the DNA, with smoking a significant risk. Cigars and shisha are known to increase the risk of developing pancreatic cancer [27].

### Prostate cancer

Hosseini M reports that shisha smoking is one of the risk factors for prostate cancer [52].

### Bladder carcinoma

Shisha smoking normally involves the use of burning charcoal, smoke inhaled by the user contains constituents originating from the charcoal in addition to those from the tobacco which is also one of the main initiating factor for developing carcinoma. In a study of 100 cases of bladder cancer, it was evaluated that 5% patients of bladder carcinoma were shisha users [53]. While about bladder cancer, a cancer-hypothesis review by Prignot et al. is quite clear: “These data indicate an increased risk, but not bladder cancer (Egypt)” [54].

### Carcinogenic effect

Shisha smoker is exposed to hundreds of potentially dangerous materials at one time. Major smoke constituents are Carbon Monoxide and nicotine [10]. Studies have shown that concentration of carcinoembryonic antigen CEA, known as marker of malignant transformation and chronic inflammation, is increased in the smoke causing a variety of cancers [6].

List of carcinogens present in tobacco smoke are: Naphthalene, Acenaphthylene, Acenaphthene, Flourne, Phenanthrene, Anthracene, Flouranthene, pyrene, Benzo[@]anthracene, Chyrsene, Benzo[b + k]fluoranthene, Benzo[a]pyrene, Benzo[g,h,i]perylene, Dibenz[a,h]anthracene, Indeno[1,2,3-cd]pyrene [55].

Health effects of shisha have been a matter of debate among researchers as different researchers having varied point of view. Composition of tobacco smoke in shisha is variable and not well standardized. It is evident that smoke emerge from water pipe contain numerous toxicants known to cause cancer [56]. Level of carbon monoxide, carboxyhemoglobbin were higher among shisha smokers than cigarette smokers or non-smokers [43]. Analysis of mainstream aerosol found that shisha smoke contains significant amount of nicotine, tar and heavy metals. In a standard heavy protocol of 100 puff of 3 sec, 2:25 mg of nicotine and 242 mg of nicotine free dry particulate were obtained. Along these, high level of arsenic, chromium and lead were found in shisha smoke. Increasing puff frequency cause increase in nicotine free dry particulate matter whereas remaining water from the bowl increases the amount of nicotine [57]. This type of data suggests shisha smoking is at least toxic as cigarette smoking. Shisha smoker may absorb large amount of these substances because of mode of smoking, depth of inhalation, length of smoking session and frequency of puffing [56]. Hazards of tar and its carcinogenicity were directly related to working temperature and not only combustion and pyrolysis [58,59]. Hookah smoking is associated with an increased risk of lung cancer in ethnic Kashmiri population with the risk being 6 times more as compared to non smokers [60]. Moreover, in a study comparing 35 healthy shisha users with 35 healthy, non-exposed controls, shisha use was associated with a significant increase in frequency of chromosomal aberrations and sister chromatid exchanges while the frequency of satellite associations and the mitotic index was significantly higher in water pipe users, relative to controls [61]. On the other side of spectrum other investigators have contradict these findings. However in striking contrast with cigarette, hookah does not generate almost no side stream smoke because of charcoal topping of the bowl and less elevated temperature so the only smoke that has been suggested be taken is the one which filtered by shisha at the level of bowl, inside water, along the level of hose, and then by smoker lung themselves but resulting smoke is expected to be less toxic than cigarette side stream smoke [6]. Study also show that shisha smoke is 3 times less concentrated than cigarette smoke as regards the particle, 74.4 × 109 for a 1000 ml hookah (machine) puff and 9.24 × 109 for a 45 ml cigarette “puff” [62]. Sajid et al. reported that as far as CEA levels are concerned heavy smokers (spending up to 6 hours per day in 3 to 8 smoking sessions of a tobacco weight equivalent to about 60 cigarettes) are very much at risk than the medium (up to 2 hrs per day in 1 to 3 smoking sessions) and light ones. Traditional hookah smoking has produce fewer carcinogenic effect than cigarette smoking but it is important to bear in mind that it still produce smoke [6]. Carcinogenicity of shisha smoking is a matter of debate among researchers, its just because of misquotations and misrepresentations in recent reviews in past researches and reviews. Situation is mainly because of three reasons, (1) the use of a neologism (“Shisha”) which has led to reduce a strikingly complex cultural and chemical variety to a theoretical “standard” object; (2) publication bias, which means that the most relevant background and cutting-edge literature was apparently dismissed; and, (3) most importantly, confusion about the chemical composition of hookah smoke (vs. cigarette smoke) [63].

### Keratoacanthoma and squamous cell carcinoma of lip

EI-Hakim IE reports that there is significant association between shisha smoking and squamous cell carcinoma and keratoacanthoma of lip [64].

### Risk of infection due to sharing

It was evaluated that the practice of sharing a water-pipe mouth piece poses a serious risk of transmission of communicable diseases including Tuberculosis and Hepatitis [27,65]. The water pipe and the water inside the Shisha apparatus can become an abode to the bacteria such as those causing TB; which can result into the spread and transmission of the disease. It is through pipe sharing with someone with pulmonary TB, which leads to a great risk of TB transmission [66].

### Strenght and limitations

The overall data that our study spans are consistent with water pipe use being associated with deleterious effects on health, but most of the source-studies involved small samples. Our study successfully provides more than sufficient material which makes a strong foundation for the detailed Meta analysis, as there always remain such resources without our reach and an impeccable search of every single article in the wide world research resources is yet to be made.

Furthermore this study also provides the basis for decision making about areas which may be open for future primary research.

### Conclusion

It is stated that “The best way to avoid the risk of those types of cancer associated with tobacco use, and particularly with cigarettes smoking, is to stop smoking entirely. In view of the fact that man may not always accomplish this objective, research efforts towards reducing the experimentally established tumorigenicity of smoking products should be vigorously continued” [67]. The review makes it crystal clear that there is a lack of knowledge regarding the hazardous effects of shisha use and its harmful effects are lurking behind a thick haze of strong misconceptions. Its use has been the triggering point of many injurious effects that culminate in the decline of the overall health status of a society. Due to rapidly increasing world wide prevalence of shisha smoking, it has now become a global issue and potential global health threat. It is necessary to address issues related to social, health and dependence aspects of water pipe use, with a focus on new ways of prevention. It also is necessary to Develop evidence based counter advertising programs to create awareness regarding its hazards.

## Competing interests

Authors declared that they have no competing interest.

## Authors’ contributions

HMA drafted the manuscript. SS, SG and WAQ critically reviewed it and makes addition. All authors declared the final version of manuscript.
